# Residual leg numbness after endoscopic discectomy treatment of lumbar disc herniation

**DOI:** 10.1186/s12891-020-03302-5

**Published:** 2020-04-27

**Authors:** Denglu Yan, Zaiheng Zhang, Zhi Zhang

**Affiliations:** 1 Orthopedics department, First People’s Hospital of Zhaoqing, Zhaoqing City, Guangdong Province 526000 People’s Republic of China; 2 Orthopedics department, People’s Hospital of Baoan, Shenzhen City, Guangdong Province 518101 People’s Republic of China; 3 Orthopedics department, Third Affiliated Hospital of Guangzhou Medical University, Guangzhou City, Guangdong Province 510150 People’s Republic of China

**Keywords:** Lumbar disc herniation endoscopic numbness

## Abstract

**Background:**

Transforaminal endoscopic discectomy was popular in the treatment of lumbar disc herniation. Previous study focuses on the leg pain of disc herniation, and little study concern the residual leg numbness after surgery. The purposes of this study were to evaluate the clinical outcomes of transforaminal endoscopic discectomy in the treatment of lumbar disc herniation with leg pain and numbness.

**Methods:**

Patients with one level lumbar disc herniation who had transforaminal endoscopic lumbar discectomy from June 2016 to July 2019 were categorized into two groups according to the leg numbness. 293 patients initially fulfilled the study criteria, and 27 patients were lost to follow-up. Of the remaining 266 patients available for analysis, 81 cases with leg numbness and pain (A group), and 185 cases with leg pain (B). Endoscopic transforaminal lumbar discectomy was performed, and the clinical outcomes of blood loss, operation times, hospital stay days, pain (Visual Analog Scale, VAS-pain), numbness (VAS-numbness), functional disability (Oswestry Disability Index, ODI), and the disk height and intervertebral foramen height were recorded.

**Results:**

All patients with pain and numbness pre-operation in group A, complain of leg numbness during or just after walking or standing not diminished after surgery in group A, and no one complain numbness after surgery in group B. The pain index and ODI score were better than preoperational in all patients (*P* < 0.01), and no significant difference between two groups (*P* > 0.05). The postoperative disk and foramen height were no significant difference compare to preoperative in all patients (*P* > 0.05), and no significant difference between two groups (*P* > 0.05). The leg numbness symptoms last longer in central disc herniation patients (10.4 ± 2.2 months) than in paracentral (6.3 ± 2.1 months) and foraminal disc herniation patients (5.6 ± 2.3 months) after surgery (*P* < 0.01).

**Conclusions:**

Based on the results of this study, transforaminal endoscopic lumbar discectomy was effective and safe procedures in the treatment of disc herniation with leg pain and numbness. The leg numbness symptoms last longer in central disc herniation patients than in paracentral and foraminal disc herniation patients after surgery.

## Background

Lumbar disk herniation is one of the most common neuropathies that presents with lower extremity symptoms. With the development of concept and technique of minimally invasive spinal surgery, many minimally invasive techniques have been developed to the lumbar disc herniation [1–7]. The current trend of evolution lumbar disc surgery has been toward transforaminal endoscopic discectomy, which is a minimally invasive treatments aimed at removing nuclear material and decompression the nerve through devices were inserted percutaneous into intervertebral discs [8, 9]. The previous studies shown that transforaminal endoscopic discectomy has the advantages of minimal invasive doesn’t damage the spinal structures, safe procedures with less trauma and faster recovery, etc. The main advantages of it was its lower extremity pain symptoms was released at once after operation, which was the reason for transforaminal endoscopic lumbar discectomy has become popular [6, 7, 10, 11].

The most common lower extremity symptoms of lumbar disc herniation is pain, but often accompanied by numbness, tingling, and sometimes a burning sensation [12–18]. In lumbar disc herniation patients, pain and numbness of the lower extremities are the most typical symptoms, which disturbed walking ability and limit their activity of daily living. After discectomy and nerve decompression, the tingling and burning sensation often released with the pain [3, 11, 12, 19]. However, the numbness always present and don’t disappear with these accompanying symptoms [2, 7, 17, 20–22]. Pain and numbness are the most typical symptoms, which disturbed walking ability and limit their activity of daily living [12, 15, 23–25]. After operation, residual leg numbness following endoscopic lumbar discectomy can lower patient satisfaction, which led to patients usually complain of leg numbness during or just after walking or standing [13, 22, 26–28]. The purpose of this study was to evaluate the clinical outcomes and efficacy of endoscopic transforaminal lumbar discectomy in the treatment of lumbar disc herniation with numbness.

## Methods

Patients with a one-level lumbar disc herniation at L4/5 or L5/S1 who underwent transforaminal endoscopic lumbar discectomy from June 2016 to July 2019 were included. The exclusion criteria included pathologic conditions of the lumbar spine (trauma, tumor, or infection) and spinal central canal stenosis causing saddle hypesthesia with bowel and bladder dysfunction. The patients were categorized into two groups according to leg numbness. Two hundred and ninety-three patients met the criteria, and 27 were lost to follow-up. Of the remaining 266 patients available for analysis, 81 patients with leg numbness and pain (group A, including 44 men and 37 women with an average age of 39.47 ± 6.14 years), and 185 patients with leg pain (group B, including 99 men and 86 women with an average age of 40.15 ± 5.39 years). There was no significant difference in A and B on age and sex distribution, older and the pain history (Table 1, *P* > 0.05).

**Table 1:** General date of patients (Means ± SD)

Group	Gender		Age	Herniation levels		Pain history	Herniation locations		
	Male	Female	(Years)	L4/5	L5/S1	(Months)	CH	PH	FH
A (81)	44	37	39.47 ± 6.14	37	44	1.25 ± 0.39	46	22	13
B (185)	99	86	40.15 ± 5.39	91	94	8.38 ± 2.47	25	133	27

Note: *CH* Central herniation, *PH* Paracentral herniation, *FH* Foraminal herniation

### Surgical procedures

**Fig. 1:**
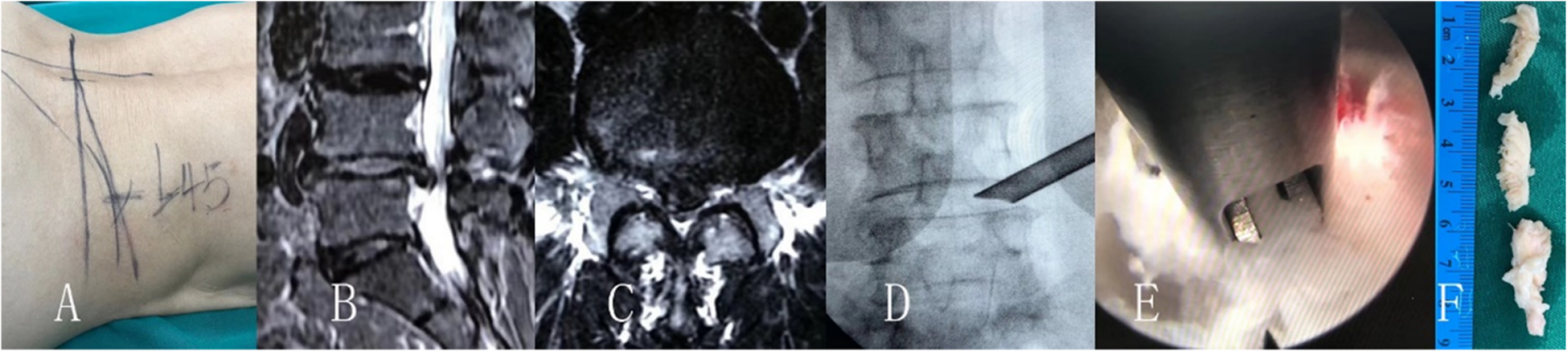
Thirty-nine years old female patient had L4/5 center disc herniation and right leg pain combine with numbness. The leg pain was disappeared after transforaminal endoscopic lumbar discectomy and nerve decompression, but the numbness was stayed until 8 months after operation. **a** show the skin marks, **b** and **c** was the MR image of the center disc herniation, **d** was the image of the endoscopic procedures, **e** show the herniation disc remove, and **f** was the removed disc herniation fragment

**Fig. 2:**
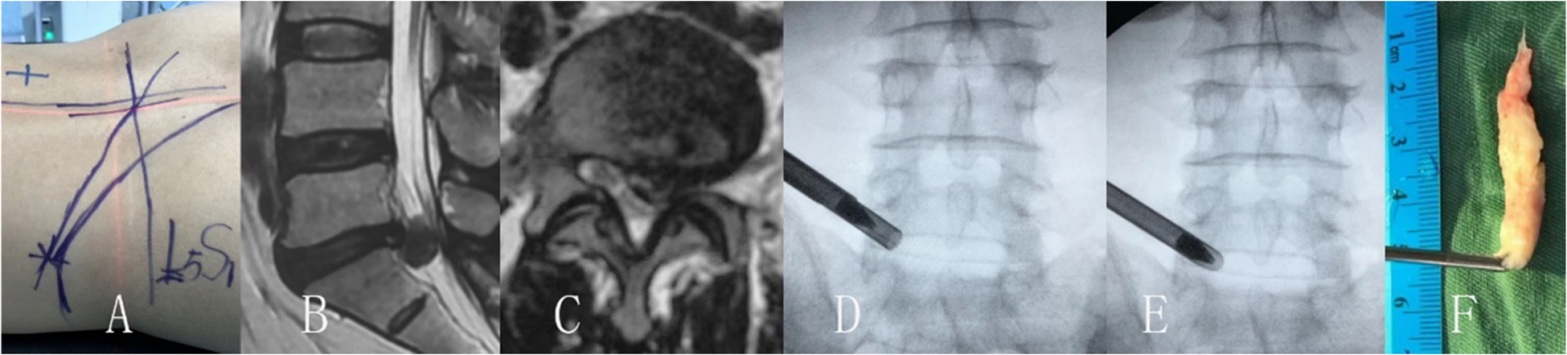
Male patient of 41 years old had L5/S1 paracentral disc herniation and left leg pain combine with numbness. The leg pain was disappeared after transforaminal endoscopic lumbar discectomy and nerve decompression, but the numbness was stayed until 6 months after surgery. **a** show the skin marks, **b** and **c** was the MR image of the left paracentral disc herniation, **d** and **e** was the image of the endoscopic procedures, and F was the removed disc herniation fragment

**Fig. 3:**
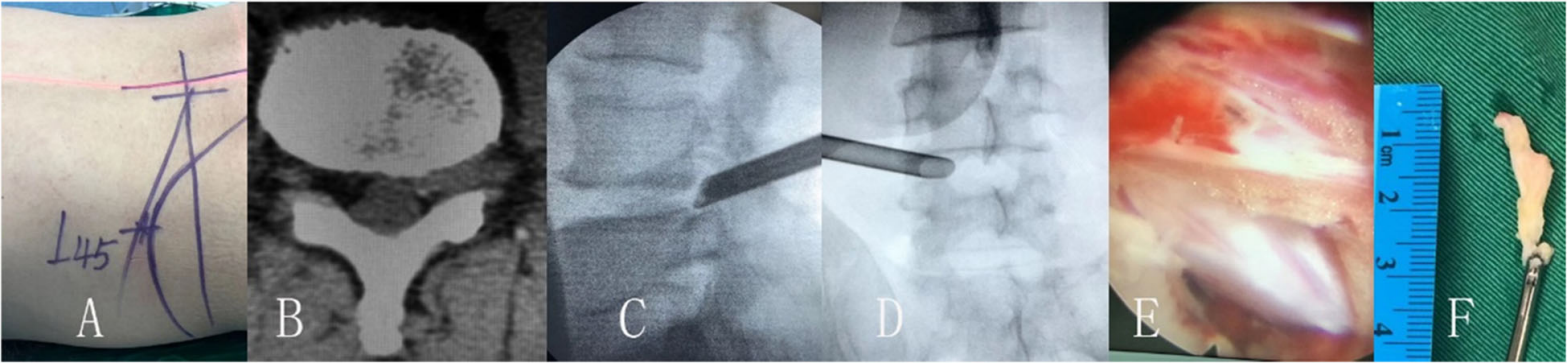
Male patient of 42 years old had L4/5 foraminal disc herniation and left leg pain combine with numbness. The leg pain was disappeared after transforaminal endoscopic lumbar discectomy and nerve decompression, but the numbness was stayed until 7 months after procedures. **a** show the skin marks, **b** was the CT image of the left foraminal disc herniation, **c** and **d** was the image of the endoscopic procedures, **e** show the nerve decompression, and **f** was the removed disc herniation fragment

The transforaminal endoscopic lumbar discectomy was performed with the patient prone on a radiolucent operating table. The entry point is planned by preoperative imaging (CT scan and MRI) which generally 10–13 cm from the midline, and the skin is infiltrated with 1% lidocaine (Figs. 1, 2, 3). A long 18-gauge spinal needle was inserted from the entry point toward the midline, in the anterior–posterior view, under intermittent fluoroscopic guidance [29, 30].

As usual performance of endoscopic discectomy, the discography was not performed (Figs. 1, 2, 3). A guide wire was then inserted and a small skin incision made around it. The dilator was inserted, and the 7 mm working sheath was inserted over the dilator. AP and lateral C-arm projections confirm the correct location of the working sheath. The endoscope is advanced through the tunnel to visualize the migrated disc herniation directly. When the disc is visualized, the use of radiofrequency probe can help shrink the fragment to be easily removed with flexible endoscopic forceps. After the herniated disc tissue was removed, the ventral epidural space is explored with an endoscopic nerve hook confirming that the herniated disc has been completely removed, and the traversing nerve root is free and totally decompression the stenosis.

Since the surgery is done under local anesthesia, the surgeon has complete communication with the patient in procedures, by checking the movements of the affected limb and patient complaining sudden and severe radicular pain during the procedure. Foraminoplasty was performed when disc herniation at L5/S1 level (Fig. 2), which removed small portions of anterolateral bone of facet articulation and its attached ligamentum flavum without interrupting the facet joint space, provided enough working space [31, 32]. A gentle hammering and twisting motion of the trephine removed anterolateral bony portions of the facet join. Then the lateral surface of the dural sac and traversing root, the posterior longitudinal ligament, and posterolateral annulus of the disc of the contralateral portion could be observed. Safely remove the herniated fragment, the ventral epidural space is explored with an endoscopic nerve hook confirming that the herniated disc has been completely removed, and the traversing nerve root is free and totally decompression the stenosis. Finally, the endoscope and working cannula are removed from the patient, and the wound is closed with a single skin suture.

### Critical of clinical outcomes

Before surgery and at the one-year follow-up, operation times, blood loss, hospital stays, pain (Visual Analog Scale, VAS-pain), numbness (VAS-numbness), functional disability (Oswestry Disability Index, ODI), and Macnab criteria were quantified in follow-up. All patients had preoperative and post-operative plain radiographs, computed tomography (CT) scans, and magnetic resonance (MR) images. The focus was to evaluate height of disk space and intervertebral foramen. The disk and foraminal height were measured using X-ray film by the computer. The disk height was the means of the anterior and posterior disk height.

### Statistical analysis

All measurements were performed by a single observer and are expressed as means ± SD. Using the SPSS 17.0 statistics software, classic t-test and chi-square test were performed. Between-group comparisons should be performed using unpaired t-tests and chi-square tests, while within-group comparisons (preoperative vs. postoperative) should be performed using paired t-tests. The threshold for statistical significance used in this study was *p* < 0.05.

## Results

The disc herniation levels were shown Table 1 and no difference distribution between two groups in the surgical level of L4/5 or L5/S1 (*P* > 0.05). The follow-up data, hospital days, average operational time and blood loose were showed in Table 2. In ends 266 cases had follow-up at least 1 year and 27 cases lost, and the follow-up rate was 88.04% (81/92) in group A, and 92.03% (185/201) in group B (*P* > 0.05). The followed time was from 13 to 18 months (average 14 months) in all patients, and average 14.82 ± 1.29 months in group A, and 14.31 ± 1.45 months in group B (*P* > 0.05). The average operational time and blood loose was 41.72 ± 11.53 min and 32.37 ± 15.59 mL in group A, and 36.83 ± 11.84 min and 29.15 ± 12.64 mL in group B (*P* > 0.05). The hospital days were no significant difference between two groups patients (3.29 ± 1.15 days in group A, and 3.47 ± 1.28 days in group B, *P* > 0.05).

**Table 2:** Follow up time, hospital days, blood loss, and operation date of patients (Means ± SD)

Group	Follow up*		Operation time*	Hospital days*	Blood loss*
	Rate	Time (Month)	(Minutes)	(Days)	(mL)
A	83.5% (81/97)	14.82 ± 1.29	41.72 ± 11.53	3.29 ± 1.15	32.37 ± 15.59
B	84.2% (239/284)	14.31 ± 1.45	36.83 ± 11.84	3.47 ± 1.28	29.15 ± 12.64

Note:*, no significant difference (*P* > 0.05)

There were no infection and no dural tear of cerebrospinal fluid leakage complications in this series cases. On the Macnab criteria, there were no patients was poor results in all patients, and there was no significant difference of good or excellent between two group patients (85.2% in group A patients, and 86.5% in group B patients, *P* > 0.05). The general, good and excellent patients was 12, 27 and 42 cases in group A, and 25, 76 and 84 cases in group B.

**Fig. 4:**
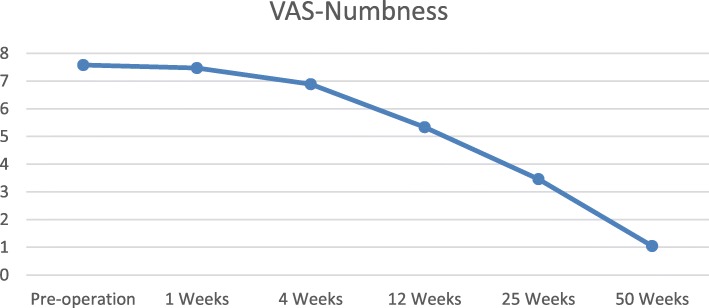
Residual leg numbness patients’ data. The pre-operation VAS-numbness scores was 7.58 ± 2.64 in group A patients. The post-operation VAS-numbness was 7.47 ± 2.72 in 1 week and 1.05 ± 0.38 in 50 weeks

Patients with pain and numbness pre-operation in group A, complain of leg numbness during or just after walking or standing not diminished after surgery (Fig. 4). However, no one complain numbness after surgery in group B, that patients no leg numbness in pre-operation. The average VAS-numbness scores were 7.58 ± 2.64 in pre-operation, 7.47 ± 2.72 in one-week post-operation, 6.89 ± 2.15 in 4 weeks post-operation, 5.34 ± 2.71 in 3 months post-operation, 3.46 ± 1.27 in 6 months post-operation, and 1.05 ± 0.38 in 12 months post-operation. The disk herniation location was significant difference between two groups (Table 1), and there were more central disc herniation patients in group A and more paracentral disc herniation in group B (*P* < 0.01). In group A, 46 patients were central herniation, 22 patients with paracentral herniation, and 13 patients was foraminal herniation. After operation, the leg numbness was lasted means 10.4 ± 2.2 months in the central disc herniation patients, released average 6.3 ± 2.1 months in paracentral disc herniation patients, and disappeared means 5.6 ± 2.3 months in foraminal disc herniation patients. The leg numbness symptoms last longer in central disc herniation patients than in paracentral and foraminal disc herniation patients after surgery (*P* < 0.01).

The VAS-pain index and ODI were better all patients (Table 3), and were significantly better than preoperative (*P* < 0.01). The VAS-pain improved from 8.29 ± 1.37 to 1.13 ± 0.46 in group A, and from 8.92 ± 1.51 to 1.18 ± 0.45 in group B. The ODI improved from 67.58 ± 14.57 to 12.41 ± 6.83 in group A, and from 63.49 ± 13.57 to 12.16 ± 6.35 in group B. There was no significant difference between two groups on the average change of VAS-pain and ODI scores (*P* > 0.05).

**Table 3:** Clinical results date of patients (Means ± SD)

Group	A^*^		B^*^	
	Preoperative	Postoperative	Preoperative	Postoperative
VAS-Pain	8.29 ± 1.37	1.13 ± 0.46	8.92 ± 1.51	1.18 ± 0.45
ODI	67.58 ± 14.57	12.41 ± 6.83	63.49 ± 13.57	12.16 ± 6.35
DH (mm)	8.68 ± 2.31	7.15 ± 2.41	8.57 ± 2.49	7.13 ± 2.18
FH (mm)	13.46 ± 1.76	11.37 ± 1.82	13.45 ± 1.63	11.39 ± 1.67

Note: ^*^, *P* > 0.05

The average disk space height and intervertebral foramen height were showed in Table 3, and there was no significant difference of postoperative compare to preoperative height (*P* > 0.05). The average disk space height and intervertebral foramen height were from 8.68 ± 2.31 mm and 13.46 ± 1.76 mm in preoperatively to 7.15 ± 2.41 mm and 11.37 ± 1.82 mm in postoperatively in group A, and from 8.57 ± 2.49 mm and 13.45 ± 1.63 mm to 7.13 ± 2.18 mm and 11.39 ± 1.67 mm in group B.

## Discussion

Degenerative lumbar disc herniation in adults that are part of the aging process may lead to compression of neurologic tissues within the spinal canal, subarticular zones or the foraminal zone, characterized by the pain of leg pain [13, 19, 33, 34]. Accurate anatomic classification of the disc herniation in the spinal canal can facilitates preoperative planning and can minimize the risk of surgical complications such as missed pathology and iatrogenic root injury [7, 23, 27]. With the minimal invasive techniques development, all sizes and types of disc herniation are possible in the hands of a skilled and experienced endoscopic surgeon [4, 14, 22]. According to the data of this case, the pain index and ODI scores were significantly better the preoperative in all patients (Figs. 1, 2, 3).

The most common symptom of lumbar disk herniation is leg pain, but this often accompanied by numbness, tingling, and sometimes a burning sensation [3, 28, 33, 35]. The transforaminal endoscopic lumbar discectomy have been demonstrated to provide a number of benefits, include less tissue trauma, preservation of normal anatomical structures, and a faster recuperative period [24, 25]. Wang et al. reported transforaminal endoscopic discectomy has excellent overall clinical effects in treating common peroneal nerve paralysis induced by lumbar disc herniation by offer sufficient decompression of the nerve root [33]. However, the accompanying symptom of numbness was difficult to diminished at once after lumbar surgery [13, 21, 26, 28]. In this series cases, residual numbness cases shown leg numbness during or just after walking or standing not diminished after surgery (Fig. 4). Some patients had residual numbness lasting more than 6 months, and some cases feel better within 6 months after surgery.

In lumbar disc herniation patients, pain and numbness of the lower extremities are the most typical symptoms, which disturbed walking ability and limit their activity of daily living [1, 35, 36]. Differences in anatomic areas of herniation affect the presenting symptoms, and herniation located canal center or foraminal can causes significant disability [11, 22, 25, 37]. As previously reported, functional neuropathies in disk herniation can be classified into nerve root compression and spinal canal central compression [3, 14, 24, 37]. The spinal central canal compression may cause saddle hypesthesia with bowel and bladder dysfunction, but these cases was excluded in this series study. Oba et al. reported lumbar disc herniation patients with longer preoperative leg numbness duration and narrow preoperative dural sac cross-sectional area showed less leg numbness improvement [28]. The results of this series cases present that most numbness cases were central disc herniation (Fig. 1). After operation, the leg numbness was lasted longer in central disc herniation patients than in paracentral and foraminal disc herniation patients. In the nerve root compression, the dominate symptom is lower extremity pain, such as radicular pain or sciatic pain. On the other hand, in the spinal central canal compression type, patients show polyradicular symptoms such as lower extremity weakness and numbness [28, 38].

Pain symptoms from lumbar disc herniation are the most common health problems in population [2, 13, 15, 21]. The endoscopic discectomy procedure was safe procedures in the situation of central disc herniation, and the neurological improvement from decompression by removed the herniated disc tissue as the results of this study. The leg pain can be effectively released by endoscopic lumbar discectomy, but leg numbness not diminished at once after surgery [11, 26–28]. Numbness of the lower extremity at rest is difficult to improve even if surgical decompression is performed, which suggests severe degeneration of the nerve roots due to chronic compression [18, 28, 38]. Although some cases numbness is resolved within 6 months, this series cases study illustrates the importance of timely to care some patients suffered from the residual leg numbness symptoms postoperatively and lasting more than 6 months.

## Conclusions

Based on the results of this study, transforaminal endoscopic lumbar discectomy was effective and safe procedures in the treatment of disc herniation with leg pain and numbness. The leg numbness symptoms last longer in central disc herniation patients than in paracentral and foraminal disc herniation patients after surgery.

## Data Availability

The datasets are available under reasonable request, please contact Yan.

## Abbreviations

VAS: Visual Analog Scale
ODI: Oswestry Disability Index
CH: Central herniation
PH: Paracentral herniation
FH: Foraminal herniation
CT: computed tomography
MR: magnetic resonance
